# Challenging the financial capture of urban greening

**DOI:** 10.1038/s41467-022-34942-x

**Published:** 2022-11-21

**Authors:** Melissa García-Lamarca, Isabelle Anguelovski, Kayin Venner

**Affiliations:** 1 Institute of Technology and Environmental Science (ICTA), Universitat Autònoma de Barcelona (UAB), Barcelona, Spain; 2 Catalan Institution for Research and Advanced Studies, (ICREA), Barcelona, Spain; 3 Basque Centre for Climate Change (BC3), Leioa, Spain

**Keywords:** Sustainability, Climate-change policy, Climate-change adaptation, Climate-change mitigation

## Abstract

Financing of urban greening has traditionally prioritized economic growth. Here the authors argue for action to ensure more socially just green financing.

## Teaser

Urban greening is critical for human health and climate adaptation and mitigation goals, but its financing tends to prioritize economic growth imperatives. This often results in elite value and rent capture and unjust greening outcomes. We argue that cities can, however, take action to ensure more socially just impacts of green financing.

## Elite financial capture of urban greening

Urban areas have long been depicted as growth machines^1^, today accounting for 80% of global Gross Domestic Product but also 75% of global carbon emissions^2^. Urban greening—referring to the physical greening and renaturing of cities through green infrastructure interventions like rail-to-trail parks, remediated waterfronts or canals, large-scale parks, or greenways and green streets—has a wealth of positive effects on mental and physical health and generates improved environmental outcomes. The many green infrastructure interventions that cities have been actively deploying over the last decade or so have climate mitigation and adaptation co-benefits like carbon sequestration, reduced urban heat island effects, and improved flooding risk management. As part of this green mission, cities are mobilizing green branding to visualize their work and compete to be the greenest city among national and international peers. Greening has also become a strategy to improve quality of life and attract private capital through direct investments or public-private partnerships which tend to increase housing prices and rents and reduce affordability^3^. Despite the latter’s negative impact on working class and racialized urban residents, the climate emergency is driving calls to policy-makers and planners to expand the scope and range of urban greening interventions, often framed as ways to unlock value and stimulate green growth.

But we must ask: Unlocking value and green growth for who? The “new” value that is generated in the process of creating urban greening comes from the metabolic relationship between capitalist societies and the biophysical world^4^. No matter how it is financed, urban greening tends to increase the value of land and property, operating as an accumulation strategy^5,6^ benefiting elite groups and reinforcing existing social and environmental inequalities. For example, research on land politics shows that extensive wetlands in Colombo, Sri Lanka have been turned into parks, canals, and real estate in recent decades, benefiting local and international investors, urban development agencies, real estate developers, and the urban upper-middle class, while low-income and marginalized populations have suffered from eviction, dispossession, and environmental hazards^7^.

In our research, we use the term “urban green grabbing”^8^ to depict how real estate developers and the financial processes surrounding them partially or completely appropriate the financial and social benefits generated by new or planned urban green amenities through building a commodity (housing developments, often large-scale ones) to be bought and sold next door. They extract extra rent, surplus value, social capital, and/or prestige from locating or financing projects adjacent to new or up-and-coming green amenities, with benefits passed onto their investors and high-end clients. Such projects take a prudential, “safe” approach to financial risk, with return on investment assured by the attractiveness of green real estate development as an asset class whose value will grow in the future^9^. Done in the name of green city-making, bolstered by an increased emphasis on urban climate adaptation and resilience, these developments often exclude working-class and racialized residents^10^.

More financing for urban greening in the context of global climate adaptation and mitigation strategies is critical, but to date it is insufficient and unevenly available^11^. Faced with budget shortfalls, cities are increasingly financing green interventions through municipal (green) bonds, tax increment financing, sale of development rights, and other direct and indirect value capture strategies^12^. These schemes embody the financialization of urban governance: city governments increasingly come to directly or indirectly rely on financial products and land markets to govern the city. Simultaneously, private capital sees public infrastructure or services as a site of accumulation, as financial interests secure revenues through the commodification and privatization of public goods^13^. Recent research has shown how green bonds, for example, tend to prioritize interventions that feed into urban economic growth logics and often reinforce existing social and environmental inequalities or create new ones^14,15^. Moreover, the financing of adaptation is so far not geared towards addressing recent or historic injustices, with recent research pointing out how financing institutions often deny credit to racialized neighborhoods exposed to climate impacts^16^.

## Green gentrification deepens urban injustices

Elite financial capture of urban greening can produce a variety of injustices. The unequal distribution of access to green infrastructure primarily occurs because of the higher land and property values new greening has been shown to produce—for the benefits of a few and the exclusion of many^17,18^. The term green gentrification is used to depict how green urban interventions attract investment and higher income and often White residents, while displacing historically marginalized groups to less green and unhealthier, climate exposed areas where they can afford to live^19,20^.

Our recent study of 28 North American and European cities identified that 17 out of 28 cities experienced these green gentrification dynamics between 1990 and 2016, whereby new green spaces—especially high-profile parks and greenways—in one time period contributed to subsequent city-wide gentrification^21,22^. For example, in Atlanta, property values increased 18%-27% more for homes located within a half-mile of the Beltline greenway than elsewhere from 2011 to 2015^23^. In Barcelona, green gentrification trends started in the 2000s and have accelerated in the past decade, with more highly educated and higher-income residents moving into traditionally working-class areas like Sant Martí while existing working-class residents had to move out. During the 2000s, the area immediately surrounding the Port Olímpic parks and Poblenou Park already saw a 26.7% and 20.5% increase in family income respectively over 5 years, compared to a 2.8% increase in the rest of Sant Martí over the same time period^24^.

Urban greening can also directly remove vulnerable residents from their neighborhoods through dwelling illegalization and land grabbing, rezoning residential neighborhoods into other uses, or labeling specific neighborhoods as high climate-risk areas, at the same time that luxury residential developments often do not have to abide by the same rules. In New Orleans, for example, the release of the Green Dot Map just a few months after Hurricane Katrina in 2005 already outlined the conversion of racialized neighborhoods such as the Lower Ninth Ward into green areas, while higher-income, yet low-lying areas, such as Lakeview were to be rebuilt^25^. Almost 20 years later, this practice of unjust environmental expropriation is still visible through contentious relocation or property buy-out programs (and subsequent re-naturing initiatives) throughout the United States. Recent research shows that the criteria and processes used in buy-outs tend to lack transparency and fairness: Low-value homes—those owned largely by working class and racialized groups—are more likely to be designated as “substantially-damaged” and thus bought out^26^. Differential treatment in the location of urban greening by race and class and protection of low-income homes is especially noticeable in the Global South^27^. In Medellín, Colombia, as the Green Belt initiative was rolled out in the early-mid 2010s, high-end residential developers in El Poblado were granted permission to build in an ecologically risky and protected area while low-income and indigenous self-built housing residents were physically, socially, and ecologically displaced in the name of nature conservation and through elite and exclusive green space use^28^.

Such examples are evidence for the idea that the financing and/or construction of green infrastructure and renaturing projects by private developers or by private-public partnerships—even when meant to be open and public—is increasingly creating privatized and enclosed spaces with unequal and limited elite access to ecological, health, or social benefits. These financial processes for new green spaces instigate new rules, norms of use, and practices that often undermine those of historically marginalized groups, and racialized groups in particular. In Barcelona, gentrification has meant that new green spaces in the Ciutat Vella (city center) have become appropriated by tourists and expat workers, mostly for entertainment and consumption purposes, in turn compromising the use of green spaces by North African and Latin American residents as well as their trust, sense of community, and place attachment^29^. When exclusive greening intersects with racialized development, cities are additionally faced with threats to emancipatory and abolitionist justice, unable to challenge deep social and racial hierarchies and guarantee the right to a “sense of place” for racialized groups^30^.

## Moving towards more socially just urban greening financing practices

We have made clear the processes and outcomes of the predominant paths to finance urban greening and the ways in which they may reinforce or create new inequalities and injustices. While there is no silver bullet to solve the problem of urban green grabbing, elite capture, and green gentrification, action can be taken so that the ecological and social benefits of urban greening investment reach populations normally left behind. If we are to avoid future “climate apartheid”^31^ that will entrench privilege and precarity within and between cities, in both the Global North and South, a shift in approach to finance urban greening and the implementation of various tools and policies is paramount.

First, financing needs to be considered as a social and ecological process, embedded in relationships and power dynamics between humans, and between humans and nature^32^. We believe emerging thinking around how to finance *reparative* climate infrastructures is a foundational approach^33^. It considers shifting capital from destructive economic sectors to ones that redress some of the inequalities, trauma, and losses generated by uneven urban development and supporting socio-natural relations of care and mutual flourishing. For example, collective community resistance in Jakarta, Indonesia has reimagined and in some cases reshaped the top-down financialized coastal and flood protection infrastructures and their financial sources, directing some funds to upgrade kampungs (informal settlements) and build protective infrastructures^34^. Along these lines, we echo calls for further research into the financial relations and tools that can support smaller scale infrastructure initiatives, especially those operating through informal economies and community-based forms of coordination, to better understand the financial processes behind more inclusive urban climate action^35^.

Another means to shift capital driving urban green growth to benefit working-class and racialized communities is through a bottom-up approach to democratize climate finance governance^36^. Incorporating grassroots engagement, subaltern forms of knowledge^37^, transparency, and accountability as core principals is urgent both globally and locally. Tools like participatory budgeting has challenged the predominant green growth paradigm in Lisbon, Portugal^38^. Lessons can also be learned from the use of explicit equity criteria in participatory budgeting institutional design in Cuenca, Ecuador, which has enabled more funds to be directed to residents most vulnerable to floods, landslides, drought and frost, all increasing in frequency due to climate change^39^.

A range of tools and policies can also be implemented by cities to regulate land use, development, and investment around green amenities^40,41^. Vacancy taxes and transfer taxes on luxury properties (Vancouver), rent controls (Berlin), development tax or linkage fee for affordable housing construction (Boston), and facilitating cooperative housing (Copenhagen, Barcelona) or community land trusts (CLTs) (Washington DC) are examples of measures that can contribute to increasing housing affordability and preventing green gentrification by controlling speculation and thus avoiding the displacement of long-term marginalized residents. More widespread adoption of these well-established tools requires bold local governments who put equity and justice concerns for marginalized groups, rather than elite profit-making interests, at the center of city planning and building processes.

**Fig. 1:**
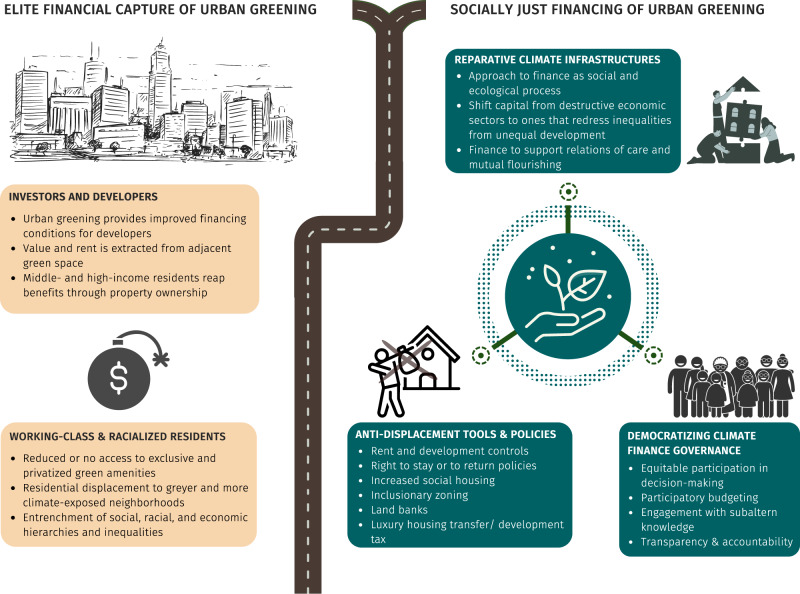
Urban greening and its financing at a crossroads. The panel on the left illustrates the path to elite financial capture, with positive effects for investors and developers and negative effects for working-class and racialized residents. In contrast, the panel on the right shows how the proposed reparative approach, anti-displacement policy tools, and democratization of climate finance governance can pave the way to socially just financing of urban greening.

In closing, the challenge of building sustainable, healthy and green cities is not simply one of increasing financing of urban greening, or closing the financing gap. Rather, financing urban greening should always be viewed in the context of how it inequitably impacts land markets and socially vulnerable  groups. We call for a shift in the way urban greening is financed from the predominant path that leads to elite financial capture to one that prioritizes equity by recognizing and seeking to meet the needs of marginalized communities. This can be achieved via a reparative approach, bold anti-displacement policy tools, and the democratization of climate finance governance (Fig. 1). While not as financially appealing as prevalent short-term profit making and economic growth incentives, the principal motivation for this new path is long-term economic, social and ecological sustainability that disrupts climate apartheid and reduces entrenched urban inequalities and vulnerabilities.
